# Notes and News

**Published:** 1902-09-13

**Authors:** 


### NOTES AND NEWS.

THE Congress of the Sanitary Institute opened at Manchester on Tuesday.

A HEALTH FARM is about to be established in Colerado for persons convalescent from consumption, who can there carry on a healthful and lucrative occupation.

HEALTH statistics for this summer show that the mortality has been low. On the other hand, minor ailments have been unusually rife, and it cannot be denied that the number of suicides is remarkably large.

THE foundation-stone has been laid of an infectious diseases hospital at Etwall for the Repton Rural District Council. The new building will consist of four detached blocks, comprising an administration, an observation, scarlet fever, and laundry block. The observation block will contain two small wards and the scarlet fever block two larger wards. The estimated cost is £6,600. The architect is Mr. Arthur Eaton, of St. James Street, Burton.

SPECIAL interest in England attaches to the Belgian Congress of Surgery now proceeding at Brussels, owing to the attention which is being paid to the subject of appendicitis. Professor Sonnenburg makes an important contribution to the discussion, giving an experience of over four hundred cases. The feeling in favour of immediate operation is very definite. The attendance of English practitioners at the Congress is very small.

A CORRESPONDENT to the daily press, commenting upon the practical ceasation of the small-pox epidemic in London, remarks that people are overlooking a great cause for thankfulness, and also the untiring and unselfish zeal of the health officers. We fear gratitude will be still more obviously overlooked in grumblings, when the ratepayer bears the burden of the cost of the epidemic. Perhaps the indifferent and even sceptical will say a word in favour of universal revaccination as a less expensive mode of preventing or stamping out future epidemics.

A MEMORIAL Cottage Hospital at St. Andrews has been opened by Mrs. Tait, who has furnished a ward dedicated to the memory of her son who fell in the South African war.

LORD WINDSOR has laid the foundation-stone of a convalescent home in connection with the Swansea Hospital. An anonymous donor has given £10,500 towards its establishment.

THERE is an epidemic of small-pox in Barbados. The inhabitants have shown resistance to the authorities who have been engaged in removing the patients from their dwellings, and a rict has ensued.

A TERRIBLE outbreak of ptomaine poisoning has just taken place at Derby. So far the mischief has been traced to a supply of pork from a well-known tradesman in Derby, and an investigation is taking place. One death has resulted, and several of the patients are critically ill. Upwards of 150 persons are reported as affected.

LIEUTENANT GIBBON, the only English officer who took part in the Belgian military ride, has been explaining his reasons for so doing and his methods. He says he joined the ride simply to see the foreign officers, and that he practically allowed his horse to go as it liked, and that though apparently no more tired than in the hunting field, it suddenly dropped dead.

THE Commission sent by the Royal Society to Uganda to study the sleeping sickness, is very actively at work. A sleeping sickness hospital has been installed and patients admitted. Of course a laboratory has been established, and Dr. Castellani, who with Doctors Low and Christy, constitutes the Commission, has prepared all his media for bacteriological examination. Dr. Low has examined the blood of about six hundred patients.

A "SILLY SEASON" correspondence is being carried on in the *Morning Post*, under the heading "Well-to-do Patients in Hospitals." The subject is a most important and difficult one, but it requires to be regarded from many different aspects, and it is doubtful whether at this season those whose utterances are of most value from a practical point of view will be found ready to give the public the benefit of their knowledge. The fact that the skill of the surgeon is a possession which he may put his own price upon is mostly overlooked when this subject is discussed, while the expense of the best appliances and nursing nowadays, is another point not properly estimated. If the difficulty of the nursing and maintenance of the middle class patient of small means may properly be met by the hospitals that is still no reason why the skilled specialist should place his services at the disposal of a larger class than he already benefits when tending the poor. Yet, unfortunately, it is a fact that the poor members of the middle classes are the only portion of the community who cannot readily obtain the highest medical and nursing skill.

